# Determinants of tuberculosis treatment interruption among patients in Vihiga County, Kenya

**DOI:** 10.1371/journal.pone.0260669

**Published:** 2021-12-02

**Authors:** Paul Waliaula Wekunda, Dickens S. Omondi Aduda, Bernard Guyah

**Affiliations:** 1 Department of Health, Tuberculosis, Leprosy and Lung Disease Control, Vihiga County, Kenya; 2 Directorate of Research, Innovation and Partnerships, School of Health Sciences, Jaramogi Oginga Odinga University of Science and Technology, Bondo, Kenya; 3 Department of Biomedical Sciences, Maseno University, Kisumu, Kenya; Chinese Academy of Medical Sciences and Peking Union Medical College, CHINA

## Abstract

**Background:**

Despite robust Tuberculosis (TB) program with effective chemotherapy and high coverage, treatment interruption remains a serious problem. Interrupting TB treatment means that patients remain infectious for longer time and are at risk of developing drug resistance and death. This study was conducted to identify and describe predictors of TB treatment interruption.

**Methods:**

A cohort of 291 notified TB patients from 20 selected health facilities in Vihiga County were enrolled in to the study and followed up until the end of treatment. Patient characteristics that potentially predict treatment interruption were recorded during treatment initiation using structured questionnaires. Patients who interrupted treatment were traced and reasons for stoppage of treatment recorded. Kaplan Meier method was used to estimate probabilities of treatment interruption by patient characteristics and determine time intervals. The Log rank test for the equality of survival distributions analyzed significance of survival differences among categorical variables. For multivariable analysis, Cox proportional hazard model, was fitted to identify predictors of TB treatment interruption through calculation of hazard ratios with 95% Confidence Intervals (CIs). For variable analysis, statistical significance was set at P ≤ 0.05. Reasons for treatment interruption were categorized according to most recurrent behavioral or experiential characteristics.

**Results:**

Participants’ median age was 40 years (IQR = 32–53) and 72% were male. Of the 291 patients, 11% (n = 32) interrupted treatment. Incidences of treatment interruption significantly occurred during intensive phase of treatment. Independent predictors of treatment interruption included alcohol consumption (HR = 9.2, 95% CI; 2.6–32.5, p < 0.001), being female (HR = 5.01, 95% CI; 1.68–15.0, p = 0.004), having primary or lower education level (HR = 3.09, 95% CI; 1.13–8.49, p < 0.029) and having a treatment supporter (HR = 0.33, 95% CI; 0.14–0.76, p = 0.009). Reasons for interrupting treatment were categorized as: alcoholism, feeling better after treatment initiation, associated TB stigma, long distance to health facility, lack of food, perception of not having TB and pill burden.

**Conclusion:**

TB treatment interruption was high and largely associated with patients’ socio-demographic and behavioral characteristics. These multidimensional factors suggest the need for interventions that not only target individual patients but also environment in which they live and receive healthcare services.

## Introduction

Tuberculosis (TB) remains the most prevalent infectious disease globally. In 2019, there were estimated 10 million cases TB and 1.4 million related deaths worldwide, with the more than two thirds occurring in south-east Asia (44%) and Africa (25%) [1]. Kenya envisions a TB free nation through a robust TB program, effective diagnosis and chemotherapy [2], but according to world health organization (WHO), the country remains one of the 30 high burden TB, TB/HIV and drug resistant TB countries in the world. The 2015/2016 Kenya TB prevalence survey, found an overall national TB prevalence of 426/100,000 and indicated that Kenya misses approximately 40% of people with TB annually [3]. Also, although there was an increase in treatment success rate (TSR) for all forms of TB from 82.4% for the 2017 cohort to 84% for 2018 cohort [4], these rates are still below the global TSR target of 90%. Comparatively Vihiga County reported a lower TSR of 81.6% in 2018 cohort which was attributable to among other factors, treatment interruption.

The WHO’s End TB Strategy and the United Nation’s Sustainable Development Goals (SDGs) share a common goal: to end the global TB epidemic. They include milestones and targets for a 90% reduction in TB deaths and an 80% reduction in TB incidence by 2030 compared with 2015 figures [5, 6]. Continued development of more convenient, reliable, and cost-effective means for early diagnosis, timely treatment over correct period of time is critical in reaching the End TB strategy targets. Apart from implementing directly observed therapy short-course (DOTs), Kenya has instituted additional measures including patient education, adherence counselling and substance abuse counselling during treatment initiation [7]. However, cumulative incidence of treatment interruption remains high, 4.5% among new TB cases and 8.5% among retreatments [8]. Interruption of TB treatment means that patients remain infectious for longer time and are at risk of developing drug resistance and death due to uncontrolled multiplication and dissemination of the TB bacillus [9, 10]. Moreover, TB treatment interruption poses a heavy economic burden to the community and health system [11]. Adherence to long course of TB treatment is complex, dynamic phenomenon with wide range of factors impacting on treatment taking behaviours [12]. Understanding factors associated with interruption of TB therapy provides basis for well-designed interventions towards optimal delivery of health care services and improvement of TB treatment outcomes. Previous studies have associated treatment interruption with socio-demographic, economic and behavioural factors such as male gender, age, alcoholism and substance abuse, feeling better shortly after treatment initiation, poor knowledge and financial difficulties [13–17]. Clinical factors such as patients coinfected with HIV who are not on antiretroviral therapy and patients who previously interrupted treatment have also been associated with TB treatment interruption [8, 18]. In Kenya, few studies have investigated impact of factors outside routine data such as income, education, severity of the disease, alcohol abuse and smoking. Moreover, there is little evidence on the rates, reasons and factors associated with TB treatment interruption in Vihiga County. The present study was conducted to identify and describe predictors of TB treatment interruption in Vihiga County.

## Materials and methods

### Study setting

This study was conducted among TB patients diagnosed and followed up in twenty selected health care facilities in Vihiga County, which located in Western region of Kenya and has a population of 590,013 [19]. The county comprises of seventy-one health care facilities located within four TB treatment zones; Emuhaya, Vihiga, Sabatia and Hamisi. The twenty selected health facilities, five from each TB treatment zone, account for over 85% of all TB cases diagnosed between 2014–2018. After diagnosis, all cases of TB are recorded in facility TB treatment register and patient record card after which they are notified into National Tuberculosis, Leprosy and Lung Disease Program (NLTP) electronic data base (TIBU) by sub county tuberculosis, leprosy and lung disease coordinators (SCTLCs).

### Study participants

This study involved patients who were 15 years or older, diagnosed with bacteriologically confirmed or clinically diagnosed TB and notified for treatment in the 20 selected health facilities in Vihiga County between June and December 2019. All cases of TB except TB affecting meninges or bone and drug resistant TB are treated with chemotherapy comprising of two months (intensive phase) with rifampicin (R), isoniazid (H), pyrazinamide (Z) and ethambutol (E) (RHZE) followed by four months (continuation phase) with rifampicin (R) and isoniazid (H) (RH) [7]. The drugs are administered orally through fixed dose combination and the dosage in determined by patients’ body weight. Most patients are treated on ambulatory basis; they receive drugs from the clinic and swallow them while at home on daily basis. During intensive phase of treatment, patients are required to visit their TB clinics once weekly and the schedule changes to two-weekly visit in continuation phase. In each clinical visit, various clinical parameters are observed and recorded after which TB medicines are issued to the patients. Drug sensitivity tests are performed to all eligible patients such as patients retreated for TB using molecular and phenotypic tests; health facilities that lack these tests send their samples to higher sites by contracted motorbike riders. This study excluded patients with drug resistant TB and those with TB affecting bones or meninges.

### Study design

A cohort of 291 TB patients in twenty selected health facilities in Vihiga County were prospectively included in to the study.

### Sample size calculation

The sample size was computed using the formula n = Z^2^pq/d^2^ [20]

Where:

n = the sample size required,
z = 1.96: confidence level test statistic at the desired level of significance,
p = 25.4%: prevalence of TB treatment interruption [21].
q = 1-p: proportion of patients with other TB treatment outcomes
d = 0.05: acceptable error of the mean willing to be committed.
Therefore n = (1.96 × 1.96 × 0.254 × 0.746) ÷ 0.05^2^.
The optimum estimated sample size was **n = 291**.

### Sampling procedure

Twenty high TB burden health facilities were purposively selected, five from each of the four TB treatment zones in Vihiga County. Together, the twenty health facilities contributed to over 85% of TB cases between 2014 to 2018 in the county. The sample was allocated to the four TB treatment zones and then twenty health facilities proportional to their contribution in the specified period (Table 1). Within facilities, simple random sampling method was used to select eligible patients into the study.

**Table 1:** Sample size allocation.

Treatment Zone	Percentage Contribution of TB Cases, 2014–2018	Allocated Sample size (n)	Health facilities	Percentage Contribution of TB cases, 2014–2018	Allocated Sample size (n)
**Sabatia**	14	40	Sabatia SCH	48	19
			Kegondi HC	16	6
			Givudimbuli HC	14	6
			Bugina HC	13	5
			Nadanya Dis	9	4
**Vihiga**	34	99	Vihiga CRH	60	59
			Mbale rural HC	17	17
			Vihiga HC	12	12
			Lyanaginga HC	8	8
			Iduku Dis	3	3
**Emuhaya**	26	76	Coptic Hospital	44	33
			Emuhaya SCH	23	18
			Ipali HC	13	10
			Ebusiratsi HC	12	9
			Esiarambatsi HC	8	6
**Hamisi**	26	76	Serem HC	29	22
			Kaimosi Hospital	18	14
			Tigoi HC	18	14
			Hamisi SCH	18	14
			Kaptech Dis	17	12
**Total**		**291**			**291**

*HC = Health Centre; Dis = Dispensary; SCH = Sub County Hospital; CRTH = County Referral Hospital.

Proportions extracted from TIBU with permission from NTLP.

### Data collection

Structured questionnaires were used to capture sociodemographic, behavioural and clinical factors that would potentially predict treatment interruption at initiation of treatment. The questionnaires were administered through face-to-face interviews by twenty trained clinicians, each attached to the participating health care facility. All enrolled patients were followed up during the entire treatment phases and any event of interest and or treatment outcomes that occurred was recorded. Additional qualitative data to elucidate reasons for TB treatment interruption was captured through National Tuberculosis, Leprosy and Lung Disease Program treatment interruption tracing form, which had a single open-ended question, reason for interrupting treatment.

### Variable definition

The outcome of interest for the present study was interruption of TB treatment (yes or no) during follow up. Treatment interruption outcome was assigned to patients who missed appointments for a period of at least two months and was confirmed by attending clinicians. Programmatic tracing of patients who interrupted treatment was usually initiated immediately the event was reported; community health volunteers (CHVs) used physical address and contact information previously obtained during enrolment to locate the patients [7]. The follow-up time for the study, the total time elapsed in days starting from the day TB treatment was initiated until patients complete their treatment, was 180 days. Time until the event occurred was defined as time in days to treatment interruption while censoring occurred when information about survival time of some patients was incomplete. Such patients included those who died, discontinued treatment, failed treatment or successfully completed their treatment.

Predictor variables considered for the study included socio-demographic characteristics such as sex (male or female), age of patients in years at the time of treatment initiation, patient education level categorized as primary or lower, secondary, or post-secondary and treatment supporter categorized as yes or no. A treatment supporter, is a household member, workmate or health care provider who is acceptable and accountable to the patient and provides directly observed therapy (DOT) and any other support to the patient. Behavioural factors for this study included smoking (yes or no) and alcohol consumption (yes or no). Patients who were categorized ‘yes’ for smoking included any patient smoking at the time of treatment initiation. Alcohol Use Disorder Identification Test (AUDIT) scoring system a 10-item screening questionnaire for hazardous and harmful alcohol consumption and alcohol related problems was used to assess alcohol use [22] at the time of treatment initiation with the minimum score being 8. It is noteworthy that alcohol, smoking and substance abuse counselling was performed on patients at the time of treatment initiation and subsequent follow ups. Clinical factors included clinical TB category categorised as bacteriologically confirmed (TB case with laboratory evidence of TB) or clinically diagnosed (a case of TB diagnosed by a provider without laboratory evidence), HIV status (positive of negative), type of TB (pulmonary TB (PTB) (TB affecting the lung) or extra-pulmonary TB (EPTB) (TB affecting other body parts outside the lungs) and type of patient categorized as new (a patient who has not previously been treated for TB), relapse (a patient who completed previous TB treatment but has another episode of TB) or treatment after lost to follow up (TLF) (a patient who interrupted previous TB treatment).

### Statistical analysis

Data was keyed in and analysed by *R* version 4.1.0. Standard descriptive statistics; proportions and median (interquartile range (IQR)) were calculated to demonstrate the socio-demographic, behavioural and clinical factors and characterize their distributions. Kaplan Meier (KM) estimator was used to obtain univariate descriptive statistics for time to treatment interruption, including estimation of probabilities of treatment interruption by patient characteristics, determine time intervals. The Log rank (Mantel–Cox) test for the equality of survival distributions was used to analyze the significance of survival differences among categorical variables and, the overall differences between estimated survival curves of patients by their characteristics. For multivariable analysis, Cox proportional hazard (CPH) model, was fitted to identify predictors of TB treatment interruption through calculation of hazard ratios with 95% Confidence Intervals (CIs). Before fitting the covariates into the model, proportional hazard assumption was checked by plotting Schoenfeld residuals against time to test for independence between time and residual. The covariate that violated the assumptions was stratified. For all variable analysis, statistical significance was set at P ≤ 0.05.

### Ethics approval and consent

This study was ethically approved by Maseno University Ethical Review Committee (MUERC) (Ref: MSU/DRPI/MUERC/00707/19) and the National Commission for Science, Technology & Innovation (NACOSTI) (Ref: 192517) and was conducted according to Helsinki’s declaration. Written informed consent was obtained from all participants and confidentiality was ensured throughout the study. For the participants below 16 years, the parents/guardians provided informed consent. The national guidelines and standard operation procedures were adhered to during treatment and follow up of TB patients.

## Results

The socio-demographic and clinical characteristics by treatment interruption, probability of completing treatment and survival difference of the study participants is presented in Table 2.

**Table 2:** Socio-demographic & clinical characteristics of TB patients by treatment interruption, probability of treatment completion and survival difference in Vihiga County.

Character	Category	Total number (%) or median (IQR)	Number (%) Interrupted treatment	Probability of treatment completion (95%CI)	Log rank p-value
Zone	Vihiga	101 (34.7)	9 (8.3)	0.90 (0.84–0.96)	0.81
	Emuhaya	87 (29.9)	11 (12.6)	0.86 (0.79–0.94)	
	Hamisi	63 (21.6)	8 (12.7)	0.86 (0.77–0.95)	
	Sabatia	40 (13.7)	4 (10)	0.89 (0.79–0.99)	
Sex	Male	209 (71.8)	24 (11.5)	0.87 (0.82–0.92)	0.74
	Female	82 (28.2)	8 (9.8)	0.89 (0.82–0.97)	
Age in years	Median (IQR)	40(32–53)	38.5 (32–49)	-	0.042
Education	Primary or lower	172 (59.1)	26 (15.1)	0.83 (0.77–0.89)	0.018
	Secondary	95 (32.6)	5 (5.3)	0.94 (0.89–0.99)	
	Post-secondary	24 (8.2)	1 (4.2)	0.96 (0.88–1	
Monthly income (KSH)	Median (IQR)	3000 (1500–5000)	3000 (1425–4000)	-	0.078
Treatment supporter	No	141 (48.5)	24 (17)	0.81 (0.74–0.88)	<0.001
	Yes	150 (51.5)	8 (5.3)	0.94 (0.91–0.98)	
Alcohol consumption	No	138 (47.4)	5 (3.6)	0.96 (0.93–0.99)	<0.001
	Yes	153 (52.6)	27 (17.6)	0.79 (0.73–0.87)	
Smoking	No	155 (53.3)	7 (4.5)	0.95(<0.92–0.99)	<0.001
	Yes	136 (46.7)	25 (18.4)	0.78 (0.71–0.86)	
Clinical condition	Stable	155 (53.3)	18 (11.6)	0.88 (0.83–0.93)	0.82
	Unstable	136 (46.7)	14 (10.3)	0.87 (0.81–0.94)	
Clinical TB category	Bact confirmed	227 (78.0)	24 (10.6)	0.88 (0.84–0.93)	0.48
	Clinically Dx	64 (22.0)	8 (12.5)	0.86 (0.77–0.95)	
HIV Status	Positive	101 (34.7)	10 (9.9)	0.88 (0.81–0.95)	0.97
	Negative	190 (65.3)	22 (11.6)	0.88 (0.83–0.93)	
Type of TB	PTB	269 (92.4)	30 (11.2)	0.88 (0.84–0.92)	0.91
	EPTB	22 (7.6)	2 (9.1)	0.89 (0.75–1.0)	
Type of TB patient	New	246 (84.5)	26 (10.6)	0.88 (0.84–0.93)	0.055
	Relapse	30 (10.3)	2 (6.7)	0.92 (0.83–1.0)	
	TLF	13 (4.5)	4 (30.8)	0.66 (0.45–0.99)	

IQR = Interquartile range; KSH = Kenya shillings (110 KSH = 1 US dollar); Bact = bacteriologically; Dx = diagnosed; TLF = treatment after lost to follow up.

A total of 291 TB patients were included into the present study. Of these, 34.7% (n = 101) were from Vihiga treatment zone and 72% (n = 209) were male. The median age was 40 years (interquartile range (IQR) = 32–53). A total of 59.1% (n = 172) of the patients had primary education or lower while the median income for the patients was 3000 Kenya shillings (IQR = 1500–4571).

The proportion of treatment interruption from, male patients, patients with primary or lower education, who consume alcohol, who smoke and those who previously interrupted treatment were 11.5%, 15.1%, 17.6% 18.4% and 30.8% respectively while probabilities of completing TB treatment were 87%, 83%, 79%, 78% and 66% respectively (Table 2). Furthermore, frequency of treatment interruption appeared higher among patients with lower age and lower monthly income.

### Time of treatment interruption

**Fig 1:**
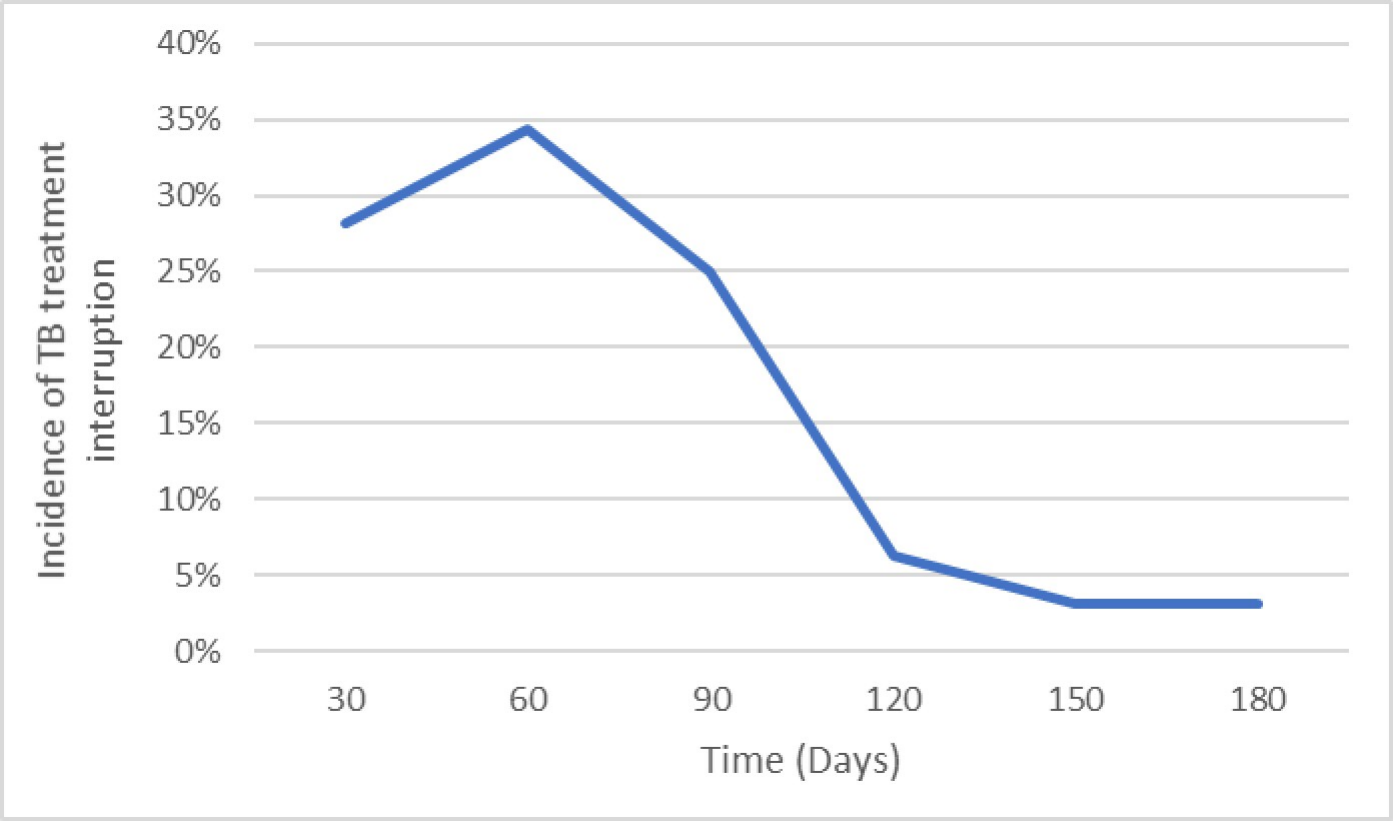
Incidence of TB treatment interruption by time.

A total of 32 TB patients (11%) interrupted treatment, while 212 (72.9%) successfully completed treatment (cured and treatment completed); 45 (15.4%) patients died, one failed treatment, and one was discontinued treatment due to adverse drug reactions. Overall, 59% (log rank p<0.001) of the incidences of treatment interruption occurred during intensive phase. The incidence of treatment interruption increased rapidly during intensive phase and by the end of third month of treatment, 88% of all incidences of treatment interruption had already occurred (Fig 1).

### Factors associated with time to treatment interruption using Kaplan Meir estimator

**Fig 2:**
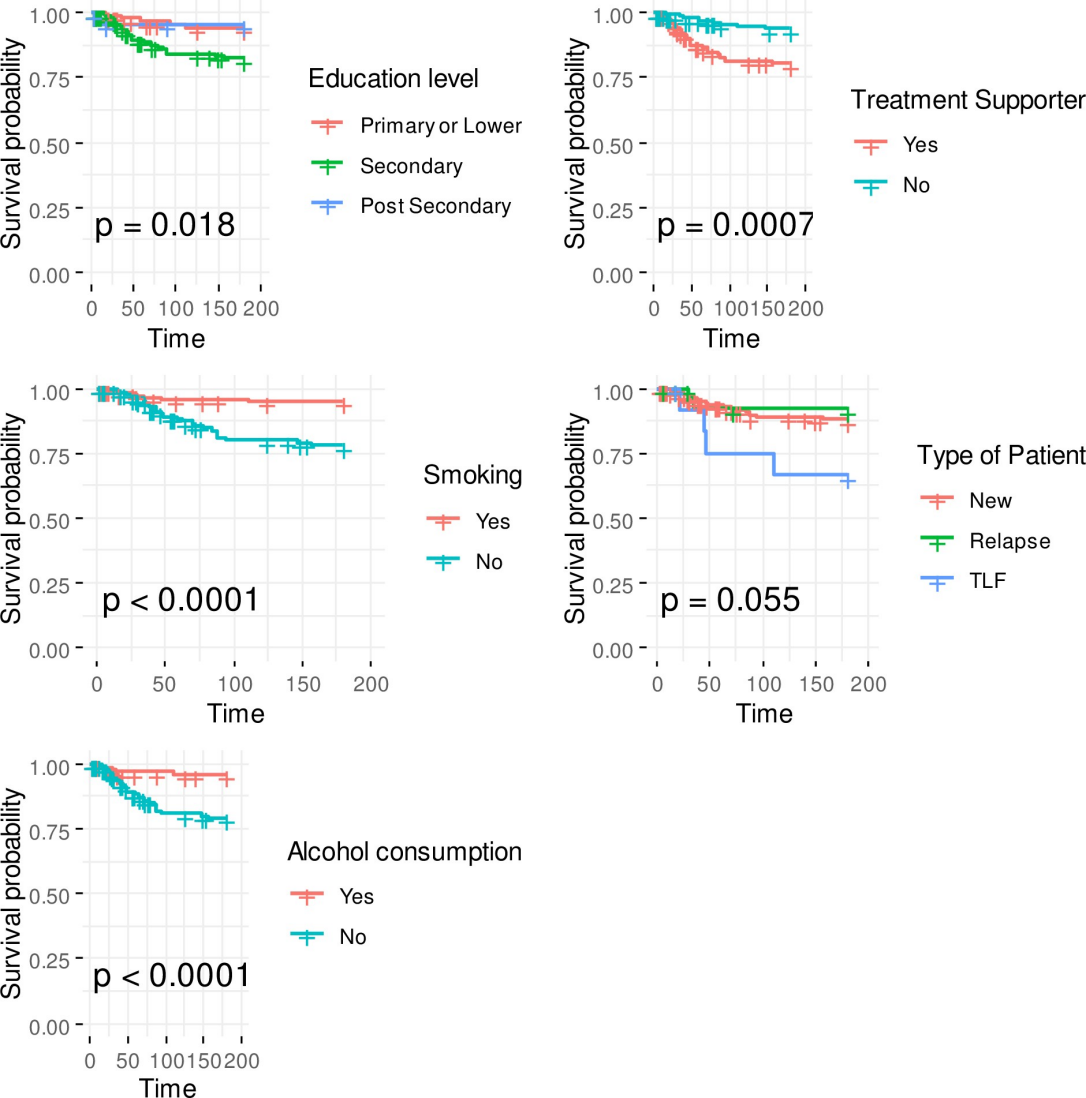
Factors associated with time to TB treatment interruption.

On univariable analysis, factors significantly associated with time to treatment interruption are presented in Fig 2. These factors included education level (Log Rank p = 0.018), treatment supporter (Log rank p <0.001), alcohol consumption (Log rank p <0.001), and smoking (Log rank p<0.001).

### Multivariable Cox regression analysis of predictors of TB treatment interruption

Before fitting the covariates into the multivariable cox model, proportional hazard assumption was checked by plotting Schoenfeld residuals against time to test for independence between time and residual. Smoking was significantly (3%) found to differ from zero at the 5% significance level, therefore, the final model was corrected by stratification of “Smoking” covariate. After simultaneously controlling for the potential predictor variables (Table 3), TB patients who consumed alcohol (HR = 9.2, 95% CI; 2.6–32.5, p < 0.001) were 9 times more likely to interrupt treatment than those who did not consume alcohol while female patients (HR = 5.01, 95% CI; 1.68–15.0, p = 0.004) were 5 times more likely to interrupt treatment compared to male patients. Also, patients with primary or lower education level (HR = 3.09, 95% CI; 1.13–8.49, p < 0.029) were 3 times more likely to interruption their treatment compared to those with secondary education. Moreover, TB patients who had a treatment supporter (HR = 0.33, 95% CI; 0.14–0.76, p = 0.009) were 67% less likely to interrupt treatment compared to patients who did not have a treatment supporter.

**Table 3:** Multivariable Cox regression analysis of predictors of TB treatment interruption among patients treated for TB in Vihiga County.

Characteristic	Category	HR^1^	95% CI^1^	p-value
Sex	Male	Reference	—	
	Female	5.01	1.68–15.0	0.004**
Age in Years	Age	0.99	0.96–1.02	0.412
Education Level	Secondary	Reference	—	
	Primary or Lower	3.09	1.13–8.49	0.029*
	Post-secondary	1.65	0.17–16.0	0.667
Monthly income (KSH)	Monthly income	1	1.00–1.00	0.797
Treatment supporter	No	Reference	—	
	Yes	0.33	0.14–0.76	0.009**
Alcohol consumption	No	Reference	—	
	Yes	9.2	2.60–32.5	<0.001***
Clinical Condition	Stable	Reference	—	
	Unstable	0.69	0.31–1.53	0.362
Clinical TB Category	Bact confirmed	Reference	—	
	Clinically Diagnosed	2.43	0.91–6.53	0.077’
HIV status	Negative	Reference	—	
	Positive	0.64	0.28–1.49	0.299
Type of TB	PTB	Reference	—	
	EPTB	0.84	0.16–4.43	0.836
Type of patient	New	Reference	—	
	Relapse	0.55	0.13–2.40	0.425
	TLF	3.11	0.96–10.1	0.058’

^1^HR = Hazard Ratio; CI = Confidence Interval; TLF = treatment after lost to follow up (treatment after treatment interruption); Bact = bacteriologically; KSH = Kenya shillings (110 KSH = 1 US dollar).

### Reasons for TB treatment interruption

Of the 32 patents who interrupted TB treatment for at least two months, 27 (84%) were found and resumed treatment, two patients were not known in the locality while three patients had migrated out. For those who were found, a single open answer question was asked “tell me the reason why you stopped taking your drugs before the required time?”. Table 4 presents each reason identified and classified, with an associated quote from a participant. The main reasons for interrupting treatment included feeling well soon after treatment initiation, alcoholism, difficulty reaching the health facility and associated stigma.

**Table 4:** Reasons the traced patients gave for interrupting TB treatment.

Reason for treatment interruption	Percentage (n = 27)	Associated quotes from patients who interrupted treatment
Felt well before treatment completion	26%	*“I stopped the drugs because I was told that my sputum tested negative at two months and I no longer cough*, *feel cold nor feel tired”*
Alcoholism	19%	*“This son of mine is never at home*. He wakes up early and go straight to chang’aa* den where he drinks until evening. *When he comes back*, *he is always too drunk to swallow medicine*. *We got tired of him”*. This was a mother referring to her son who was also present.
Difficulty reaching health facility	15%	*“My legs are weak; I am not able to walk to the facility”*
Stigma	15%	*“I recently came from Nairobi sick and weak*, *if I come to HIV clinic*, *people will say ‘he brought HIV from Nairobi”*
Perception of not having TB	11%	*“My sputum tested negative for TB; how then do you tell me that I have TB*?*"*
Pill burden	7%	*“I’m taking too many drugs*. *I’m taking ARVs*, *TB drug and drugs for hypertension*. *I had to stop taking TB drugs because they are too many and big”*
Lack of food	7%	*“I feel terrible when I swallow the medicine on empty stomach*. *I discontinued the medicine because I don’t have enough food”*

*chang’aa = Locally prepared alcoholic drink.

## Discussion

This study was conducted to identify and describe predictors of TB treatment interruption. About one in every ten TB patients interrupted their treatment, predominantly during intensive phase of treatment. The treatment interruption rate in our study is higher than national treatment interruption rate of 4.5% among new TB patients and 8.5% among retreatments [8] but lower compared to Kiambu county, Kenya (20.9%) [15], Ethiopia (21.21%) [23] and Mbarara hospital, Uganda (25%) [21]. This is consistent with a regional widespread problem. To achieve global TSR target of 90%, which is a key milestone towards achieving sustainable development and End TB strategy goals of zero TB epidemic, it is mandatory to lower the levels of TB treatment interruption. One possible reason why patients interrupt the treatment during intensive phase is that they may feel better soon after treatment initiation as revealed by more than a quarter of patients who were traced after interrupting treatment. Majority of TB bacilli are killed during the intensive phase thereby reducing clinical symptoms [24] and this may give patients false sense of being cured. This suggests the need for continuous health education and adherence counselling. Previous studies in Kenya [8, 14, 15] and other Ethiopia [17] have also demonstrated higher incidences of treatment interruption during intensive phase of treatment. Nevertheless, studies in Mbarara hospital, Uganda [21] and Gondar town Ethiopia [25] indicated that interruption of TB treatment occur during continuation phase. This may be attributable to differences in patient characteristic, operational definition of treatment interruption and study design. Early identification and mitigation of risk factors for TB treatment interruption is important to reduce incidences of treatment interruption among TB patients.

Our study clearly indicate that alcohol consumption is a significant risk factor for treatment interruption and is one of main reasons cited by patients who interrupted TB treatment. Patients who consume alcohol are likely to forget taking their drugs especially when they are drunk. Besides, alcohol and anti-TB medication are likely to interact causing undesirable effects that may cause patients to stop taking TB drugs [24]. Previous studies in Nairobi, Nakuru and Kericho [13, 14] as well as systemic reviews in African region [26, 27] have similarly demonstrated that alcohol use is a major risk factor for TB treatment interruption. Of concern is that alcohol is consumed by a group of people often in enclosed places [28]. This confers a great risk of prolonged community TB transmission since the enclosed places are usually overcrowded and poorly ventilated. Alcohol, smoking and substance abuse counselling is routinely provided for all eligible patients when starting TB treatment [7], but there is need to explore its uptake, effectiveness and challenges in context of TB treatment outcomes.

Several studies [8, 14, 15] have associated TB treatment interruption with male gender, possible reasons being cited as males’ mobility and poor health seeking behaviour. Several other studies [13, 18, 21, 25, 29] have shown that there is no sex deference in TB treatment interruption. Our study associated female gender with TB treatment interruption. The difference in study findings might be attributable to sample size, socio-cultural differences such as education level and differences in patients’ behaviours such as alcohol consumption and smoking. In this study there is very strong gender difference in alcohol consumption and smoking which are more prevalent in male than female. Possible confounders in our study were accounted for through restriction by age and duration of TB treatment and by use of multivariable Cox regression. A larger sample is desired to elucidate the sex-specific difference in the risks associated with TB treatment interruption.

Our study has demonstrated that lower education level is associated with interruption of TB treatment. Studies conducted in Kenya [13, 14], Sudan [30] and Ethiopia [23] have similarly associated illiteracy with TB treatment interruption. These findings are consistent with known facts about education as a social determinant of disease and related risks [31]. Higher formal education is likely to influence lifestyle, psycho-social skills and values that may protect individuals against poor health behaviours such as treatment interruption. Patients with less education might have less understanding about TB disease and importance of adherence to treatment. For the benefit of all patients, it is imperative that clinical messages are simplified to suit patients with lower education level. Nyi and Chuah from Malaysia reported that educational level is not associated with TB treatment interruption [32]. This contrast may be due to differences in study design.

Our study found that having a treatment supporter is a preventive effect against interrupting TB treatment. This indicates that patients who interrupted treatment did not have a treatment supporter. Studies in rural and urban settings in Kenya [33], Hadiya Zone, South Ethiopia [34], and Windhoek District, Namibia [35] similarly found that treatment supporters are critical mitigation for treatment interruption. This is probably because treatment supporters directly observe patients while they take their medication, are likely to provide social support and remind patients about their clinic appointment. Although interventions using DOT have shown variable effects on TB treatment outcomes [36], treatment supporters selected by patients and community have been shown to be more appropriate in developing countries where TB burden is high because they are often available and are more cost effective [37–39].

Reasons that patients gave for interrupting treatment are largely socio-economic and behavioural risk factors that suggest the need for a well-designed social support network, health promotion and education strategies. Using a person-centred approach is widely advocated to enable design interventions that are tailored to meet ongoing patient treatment monitoring and psychosocial support needs. However, previous studies in Sub Saharan Africa [40, 41] have indicated that poor healthcare worker interpersonal communication and messaging of medical instructions to patients are common cause of lack of treatment compliance and access to care, hence the need for integrated care approach that meet multi-dimensional patient needs.

### Strength of study

The prospective design of this study has allowed recording of potential predictors of treatment interruption from the beginning of the study in a routine health care setting. As a result, the risk of recall bias and record errors which is an inherent problem of retrospective and cross-sectional study designs, is taken into account. We were also able to assess factors not often recorded in electronic database. Moreover, quantitative and qualitative approaches in the study enabled understanding of attitudes and perception of patients who interrupted TB treatment.

### Study limitation

We acknowledge a few limitations in our study. First, we included only patients from high TB burden health facilities and this might cause slight bias. Secondly, alcohol consumption and smoking were self-reported at the beginning of treatment and this may have led to underestimation or overestimation of prevalence of alcohol abuse and smoking among our study participants. Moreover, is appears during the study we did not account for alcohol abuse and smoking counselling.

## Conclusion and recommendations

The rate of TB treatment interruption is higher than expected in Vihiga County, and frequently occur during intensive phase of treatment. The main predictors of treatment interruption are alcohol consumption, being female and having primary or lower-level education and having treatment supporter. Furthermore, main reasons for interrupting treatment seems to be sense of feeling well soon after starting treatment, alcohol abuse and stigma. These multidimensional factors suggest the need for interventions that not only target individual patients but also environment in which they live and receive healthcare services.

## Supporting information

S1 Data (XLSX)
